# Peripheral myelin protein 2 is underexpressed in early-onset colorectal cancer and inhibits metastasis

**DOI:** 10.3389/fmolb.2025.1610003

**Published:** 2025-06-05

**Authors:** Zhiyu Yu, Sen Wang, Peng Xu, Cheng Zhang

**Affiliations:** ^1^ Department of General Surgery, General Hospital of Northern Theater Command, Shenyang, Liaoning, China; ^2^ Jinzhou Medical University, Jinzhou, Liaoning, China

**Keywords:** early-onset colorectal cancer, weighted gene co-expression network analysis, metastasis, machine learning, PMP2

## Abstract

**Background:**

The occurrence and fatality rates of early-onset colorectal cancer (EOCRC) are increasing, with metastasis being one of the primary causes of the high mortality rate in EOCRC patients. Currently, there is a shortage of biomarkers for diagnosis and targets for treatment with optimal efficacy and reliability. This study aims to identify biomarkers associated with metastasis to provide effective diagnostic strategies for EOCRC patients.

**Methods:**

Expression datasets were retrieved from The Cancer Genome Atlas (TCGA) database and analyzed for differentially expressed genes (DEGs). Subsequently, weighted gene co-expression network analysis (WGCNA) was performed to identify gene modules associated with EOCRC metastasis. Feature genes were selected using machine learning and tdiagnostic performance of these genes was assessed with receiver operating characteristic (ROC) curves. We validated peripheral myelin protein 2 (PMP2) expression levels in EOCRC tissues at the molecular and protein levels using quantitative reverse transcription polymerase chain reaction (qRT-PCR) and immunohistochemistry (IHC). Finally, the invasive and migratory capabilities of PMP2 were assessed in this study using Transwell assays.

**Results:**

The analysis identified 1,411 DEGs in EOCRC and 3,434 DEGs in late-onset colorectal cancer (LOCRC). Subsequently, WGCNA filtered out module genes specifically associated with metastasis in EOCRC, leading to 44 genes. We identified four feature genes by analyzing these genes using machine learning algorithms and taking the intersection: LINC02268, AC092652.1, GRIK1, and PMP2. The ROC curve indicated that these four genes are vitally involved in EOCRC. Further, qRT-PCR and IHC highlighted that PMP2 was downregulated in EOCRC tumor tissues compared to normal tissues. Moreover, Transwell assays revealed that PMP2 inhibited the invasion and migration of EOCRC.

**Conclusion:**

PMP2 has low expression in EOCRC tissues and inhibits EOCRC metastasis, serving as a potential biomarker and therapeutic target for EOCRC treatment.

## 1 Introduction

Colorectal cancer (CRC) is a common malignant tumor of the digestive system and is globally the third most prevalent type of cancer (Saraiva et al., 2023). Early-onset colorectal cancer (EOCRC) refers to CRC diagnosed in individuals under the age of 50, the incidence of which is rapidly rising (O’Sullivan et al., 2022). Compared with late-onset colorectal cancer (LOCRC), EOCRC is more invasive and shows a higher risk of metastasis (Uy et al., 2004; Dozois et al., 2008). Further, patients with EOCRC are usually diagnosed at an advanced stage of the disease. The pathological types in these patients are mostly mucinous carcinoma and signet ring cell carcinoma, and the tumor differentiation degree is generally low. These patients typically present with a more advanced clinical stage, higher levels of tumor markers, lymph node metastasis (N) and distant metastasis (M). Metastasis is the main cause of poor prognosis in EOCRC patients (Wang et al., 2015; Vuik et al., 2021; Yimin et al., 2023). This study was aimed at identifying feature genes associated with EOCRC metastasis and assessing their biological functions and underlying processes.

In this study, we focused on the peripheral myelin protein 2 (PMP2) gene, a fatty acid-binding protein (FABP) that plays a key role in lipid metabolism (Majava et al., 2010). Previous studies have shown that other members of the FABP family are closely associated with cancer development and progression. Further, they may play a role in tumor development by modulating lipid metabolism and cell signaling pathways (McKillop et al., 2019). Given these findings, our study aimed at exploring PMP2 expression pattern in EOCRC and its relationship with clinical features using bioinformatics analysis.

Identifying reliable biomarkers is crucial for the treatment of patients (Ye et al., 2025). In this study, we used weighted gene co-expression network analysis (WGCNA) and machine learning techniques to obtain feature genes from multidimensional datasets and better comprehend EOCRC pathogenesis (Langfelder and Horvath, 2008; Deo, 2015; Kumar et al., 2021). We used WGCNA to analyze genes connected with EOCRC metastasis, followed by functional enrichment analysis. We screened feature genes using the least absolute shrinkage and selection operator (LASSO) and support vector machine-recursive feature elimination (SVM-RFE). We conducted immune cell infiltration analysis, immune checkpoint analysis, and receiver operating characteristic (ROC) curve evaluation using these feature genes. Finally, we corroborated the differential expression levels of feature genes and their specific biological functions in EOCRC, to better provide diagnostic markers and therapeutic targets for the malignancy.

## 2 Materials and methods

### 2.1 Data sources and analyses

We first downloaded the RNA-seq expression data for EOCRC (<50 years old) from The Cancer Genome Atlas (TCGA) database (76 tumor and 7 paired non-tumor samples). We next downloaded the RNA-seq expression data for LOCRC (≥50 years old), which included 566 tumor and 44 paired non-tumor samples. We screened for DEGs using the “edgeR” package in R, with the screening criteria being |log2FC| > 2.5, P < 0.01. The clinical information incorporated in the analysis were N and M stages. After removing samples with missing clinical information and duplicate samples, 66 EOCRC tumor samples remained for subsequent analysis, while 478 LOCRC samples remained.

### 2.2 Weighted gene co-expression network analysis construction

WGCNA reveals the functional links among genes by constructing gene co-expression networks to group genes with comparable expression profiles into common modules. We used the “WGCNA” package in R for analysis, which identified modules and genes associated with EOCRC and LOCRC metastasis.

### 2.3 Functional analysis

We used the “clusterProfiler” package in R for functional analysis to confirm the potential roles of the identified genes. Disease Ontology (DO) and Gene Ontology (GO) analyses were conducted for genes associated with EOCRC metastasis. Additionally, we performed the Kyoto Encyclopedia of Genes and Genomes (KEGG) analysis to study the enrichment of gene signaling pathways. P < 0.05 was considered to be statistically significant.

### 2.4 Machine learning-based analysis

The genes associated with EOCRC metastasis were analyzed with the “glmnet” and “kernlab” packages in R to identify feature variables by LASSO regression and SVM-RFE machine learning algorithms, respectively. We also used the online tool Venny 2.1.0 to conduct an intersecting analysis of the results to select feature genes only related to EOCRC metastasis.

### 2.5 Receiver operating characteristic curve analysis

The ROC curve was constructed with the “pROC” package in R to evaluate the potential of feature genes as biomarkers and their association with disease occurrence and progression.

### 2.6 Immune infiltration analysis

The “GSVA” package in R was utilized to evaluate the association between immune cell infiltration and the feature genes related to EOCRC metastasis. We obtained distribution patterns of the feature genes related to EOCRC metastasis for 28 types of immune cells using the ssGSEA algorithm. The immune cells related to the feature genes of EOCRC metastasis were screened using P < 0.05.

### 2.7 Immune checkpoint analysis

The “corrplot” package in R was used for immune checkpoint analysis to understand the immune regulatory mechanisms and gene interactions.

### 2.8 Cell culture and transfection

LOVO cells (EallBio, CA) were maintained in F-12K complete medium and HCT116 cells (EallBio, CA) were maintained in McCoy’s 5a complete medium. Cells were cultivated in a humidified incubator with an atmosphere containing 5% CO_2_ at 37°C. Once the cells reached 40% confluence, they were transfected with LipofectamineTM 2000 (Invitrogen, United States) and siRNA targeting PMP2 with a negative control siRNA to reduce PMP2 expression. After transfection for 6 h, fresh culture medium was added to replace the old medium, the cells were cultured for 24 h, and the cells were collected for invasion and migration experiments.

### 2.9 Quantitative reverse transcription polymerase chain reaction

TRIzol (Invitrogen, United States) was used to extract RNA from clinical tissues collected from the General Hospital of the Northern Theater Command. The concentration of RNA was determined using the NanoDrop 2000 (Thermo Fisher Scientific, United States). RNA was reverse transcribed into complementary DNA using the Prime-Script RT reagent kit (TaKaRa, Japan). Subsequently, qRT-PCR was done using Ex-Taq (TaKaRa, Japan) and expression levels were determined with the 2^−ΔΔCT^ method. The primer sequences are given below: PMP2 forward, 5′-GACGATTACATGAAAGCTCTGGG-3′; PMP2 reverse, 5′-TGCACTTGATTCAGTGATCCTCT-3′; GAPDH forward: 5′-GGAGCGAGATCCCTCCAAAAT-3′; GAPDH reverse: 5′-GGCTGTTGTCATACTTCTCATGG-3′.

### 2.10 Migration and invasion assays

To determine the migration of LOVO and HCT116 cells, 1 × 10^5^ cells were suspended in medium containing 2% FBS and plated in the upper chamber, while the lower chamber medium contained 20% FBS. After incubation for 24 h, cells were fixed with methanol, stained with crystal violet, and counted under an inverted microscope. In the invasion assay, 1 × 10^5^ cells were suspended in medium with 2% FBS and plated in the upper chamber coated with Matrigel (Corning, NY, United States), while the lower chamber medium contained 20% FBS. After incubation for 24 h, cells were fixed with methanol, stained with crystal violet, and counted under an inverted microscope. Each experiment was repeated at least three times.

### 2.11 Immunohistochemistry experiments

The EOCRC and adjacent normal tissue slices were subjected to dewaxing, hydration, and rinsed with pure water. Antigen retrieval was done by placing the sections into a citrate buffer solution and heating them in a microwave for 10 min before cooling them to room temperature. The endogenous peroxidases were inactivated by treating sections with a 3% hydrogen peroxide solution for 10 min. The sections were then incubated with anti-PMP2 primary antibodies (1:100; Proteintech Group, China) overnight at 4°C. Subsequently, the sections were incubated with secondary antibodies at 37°C for 30 min. After using the DAB solution for color development, the slices were stained with hematoxylin, dehydrated and sealed with resin. Finally, the sections were observed and photographed under a microscope (Nikon, Japan).

### 2.12 Clinical tissue sample collection and processing

In this study, 49 EOCRC specimens and adjacent normal tissue were collected from patients who underwent surgery at the General Hospital of the Northern Theater Command. None of the patients received neoadjuvant therapy before surgery. We obtained informed consent from each patient who participated in the study. The Ethics Committee of the Northern Theater General Hospital approved the current work (Y (2022) 063).

### 2.13 Statistical analysis

Statistical analysis was performed using R software (version 4.1.3). The two-tailed Student’s t-test was done to evaluate differences between groups, with statistical significance at P < 0.05.

## 3 Results

### 3.1 Research flowchart

**FIGURE 1:**
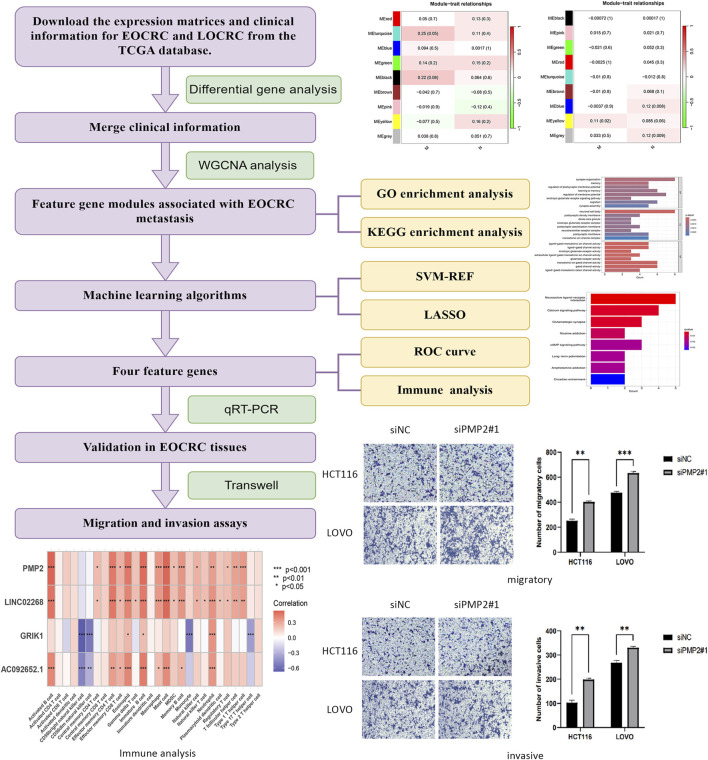
Flowchart of the study.

The methodologies employed in this study have been shown as a flowchart in Figure 1.

### 3.2 Differentially expressed gene screening and weighted gene co-expression network analysisconstruction

**FIGURE 2:**
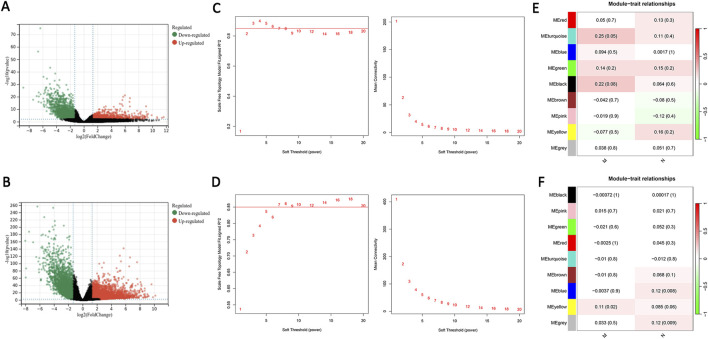
DEGs screening and weighted gene co-expression network analysis construction. **(A)** Volcano plot of DEGs in EOCRC. **(B)** Volcano plot of DEGs in LOCRC. **(C)** The soft threshold was β = 4 in EOCRC. **(D)** The soft threshold was β = 7 in LOCRC. **(E)** The relationship between the modules and clinical traits in EOCRC is shown in the heatmap. **(F)** The relationship between the modules and clinical traits in LOCRC is depicted in the heatmap. Key: DEGs: differentially expressed genes; EOCRC: early-onset colorectal cancer; LOCRC: late-onset colorectal cancer.

The EOCRC and LOCRC gene expression matrices and clinical data were downloaded from the TCGA database. We selected DEGs using the criteria |log2FC| > 2.5, P < 0.01. We identified 1,411 DEGs in EOCRC, and 3,434 DEGs in LOCRC (Figures 2A,B). The association between these DEGs and clinical traits was assessed by individual WGCNA for EOCRC and LOCRC, with the soft thresholds set at 4 and 7, respectively, ensuring the biological significance of network construction (Figures 2C,D). The M and N were selected as clinical traits in the analysis to identify gene modules related to metastasis. We found a positive correlation between the cyan module and M in EOCRC, with a correlation coefficient of 0.25. In LOCRC, the blue and gray modules were positively correlated with N, and a positive correlation emerged between the yellow module and M, with correlation coefficients of 0.12, 0.12, and 0.11, respectively. The modules significantly related to M and N based on these findings were selected for further analysis (Figures 2E,F).

### 3.3 Screening of key module differentially expressed genes and functional analysis

**FIGURE 3:**
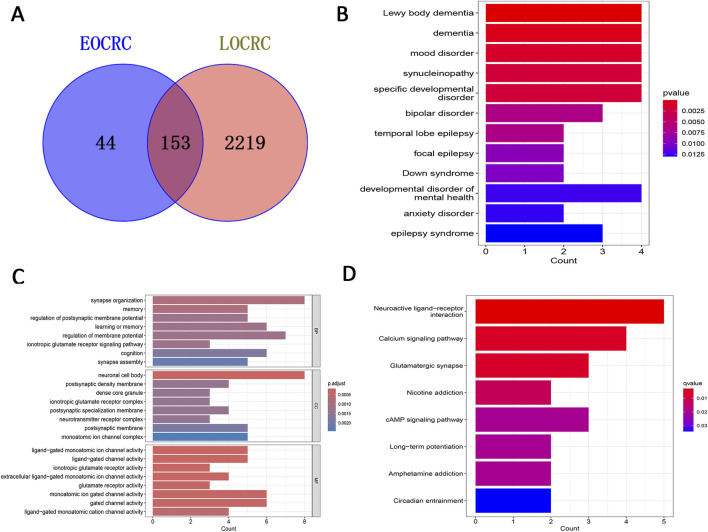
Screening of key module DEGs and functional analysis. **(A)** Venn diagram displaying feature genes only associated with EOCRC metastasis. **(B)** DO analysis. **(C)** GO analysis. **(D)** KEGG analysis. Key: DEGs: differentially expressed genes; EOCRC: early-onset colorectal cancer; DO: Disease Ontology; GO: Gene Ontology (GO); KEGG: Kyoto Encyclopedia of Genes and Genomes.

A Venn diagram was employed to identify 44 genes specifically related to EOCRC metastasis (Figure 3A). We performed functional analysis on these genes to delve deeper into their biological roles and mechanisms of action. The DO analysis results suggested these genes are primarily linked to Lewy body dementia and mood disorders (Figure 3B). Biological processes (BP) were mainly related to synaptic organization and regulation of membrane potential, Cellular components (CC) were associated with neuronal cell bodies and postsynaptic membranes and Molecular Functions (MF) were mostly linked with ion channel activity (Figure 3C). KEGG analysis highlighted that these genes predominantly participated in neuroactive ligand-receptor interaction and calcium signaling pathways (Figure 3D).

### 3.4 Machine learning-based screening and subsequent validation of feature genes

**FIGURE 4:**
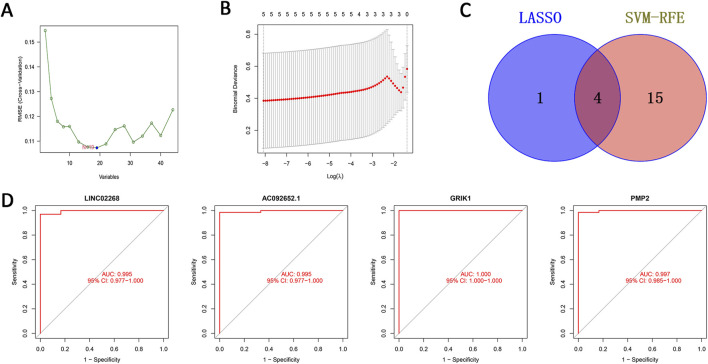
Machine learning-based screening of feature genes and subsequent validation. **(A)** Validation of biomarker feature gene expression through the SVM-RFE algorithm. **(B)** Feature selection adjustment through the LASSO algorithm. **(C)** Venn diagram of the two machine learning algorithms. **(D)** ROC curves of the four feature genes. Key: ROC: Receiver Operator Curve; SVM-RFE: support vector machine-recursive feature elimination; LASSO: least absolute shrinkage and selection operator.

We utilized two methods to highlight feature genes related to EOCRC metastasis. While the SVM-RFE technique identified 19 genes, the LASSO regression analysis filtered out five genes. (Figures 4A,B). We then performed an intersecting analysis of the results from both approaches using a Venn diagram and ultimately selected four feature genes: LINC02268, AC092652.1, GRIK1, and PMP2 (Figure 4C). We plotted their ROC curves and calculated the area under the curve (AUC) values to be 0.995, 0.995, 1.000, and 0.997, respectively. These high AUC values indicate the high accuracy of these genes as disease feature genes to be effective diagnostic biomarkers potentially (Figure 4D).

### 3.5 Immune analysis of feature genes and immune checkpoint analysis

**FIGURE 5:**
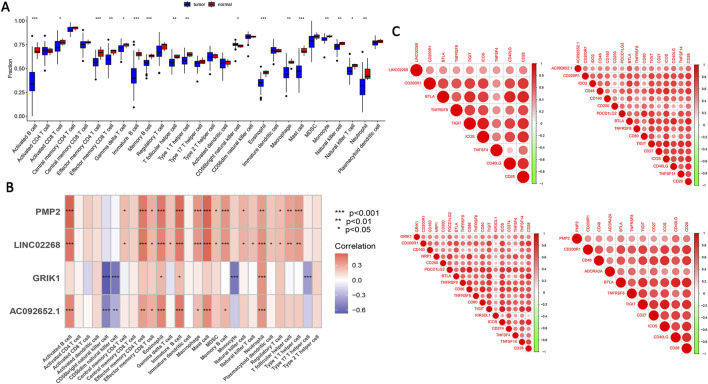
Immune analysis of feature genes and immune checkpoint analysis. **(A)** Variations in immune infiltration were observed between the EOCRC patient samples and healthy individuals. **(B)** The association between the four feature genes 28 types of immune cells. *p < 0.05, **p < 0.01, ***p < 0.001. **(C)** The association between the four feature genes and immune checkpoint genes. Key: DEGs: differentially expressed genes; EOCRC: early-onset colorectal cancer.

We further analyzed the differences in immune infiltration between EOCRC and control samples. Significant differences were seen in activated B cells, activated CD8 T cells, effector memory CD4 T cells, effector memory CD8 T cells, gamma delta T cells, immature B cells, memory B cells, T follicular helper cells, Type 1 T helper cells, CD56bright natural killer cells, eosinophils, macrophages, mast cells, monocytes, natural killer cells, natural killer T cells, and neutrophils (Figure 5A). These results indicated significant alterations in composition and functional status of immune cells in the EOCRC tumor microenvironment, suggesting the possible role of immune cells in EOCRC occurrence and progression. We utilized the ssGSEA algorithm to assess the relationship between the four feature genes and immune cells in EOCRC. we found that the four feature genes were closely related to activated B cells, eosinophils, immature B cells, mast cells, and neutrophils, suggesting their putative role in disease progression by immune microenvironment modulation (Figure 5B). Additionally, immune checkpoint analysis documented a positive correlation between the four feature genes and immune-related genes (Figure 5C).

### 3.6 PMP2 is underexpressed in early-onset colorectal cancer and inhibits the cell invasion and migration

**FIGURE 6:**
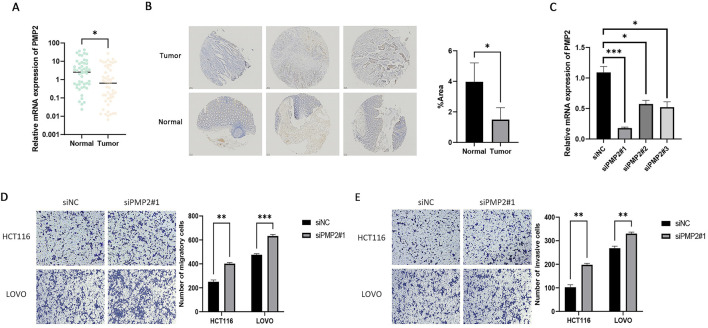
PMP2 is underexpressed in EOCRC and inhibits the invasion and migration of EOCRC. **(A)** Comparison of PMP2 mRNA levels in EOCRC tissues vs. adjacent non-tumor mucosa. *p < 0.05. **(B)** Comparison of PMP2 protein levels in EOCRC tissues vs. adjacent non-tumor mucosa. **(C)** The transfection efficiency of siPMP2#1, siPMP2#2, and siPMP2#3 in HCT116 cells was determined using qRT-PCR. *p < 0.05, ***p < 0.001. **(D)** Assessment of the migration ability of HCT116 and LOVO cells following PMP2 knockdown. **(E)** Assessment of the invasion ability of HCT116 and LOVO cells following PMP2 knockdown. **P < 0.01, ***P < 0.001. Key: EOCRC: early-onset colorectal cancer.

We selected PMP2 for subsequent experiments as there are no current reports on the association between PMP2 and CRC. We obtained 49 paired EOCRC and neighboring normal tissues from the Northern Theater Command General Hospital. The qRT-PCR demonstrated that PMP2 levels were markedly lower in EOCRC tissues compared to normal samples (Figure 6A). Subsequently, we used IHC to analyze PMP2 expression levels in three pairs of EOCRC and adjacent normal samples and found that PMP2 is underexpressed in EOCRC (Figure 6B). Subsequently, three different siRNAs were used to knock down PMP2 in HCT116 cells to verify the siRNA knockdown efficiency. qRT-PCR results showed significantly decreased PMP2 expression in HCT116 cells transfected with siPMP2#1/2/3 compared to the siNC group (Figure 6C). Additionally, the lowered PMP2 expression significantly enhanced the migratory and invasive abilities of HCT116 and LOVO cells than the siNC group (Figures 6D,E). These results indicate that PMP2 knockdown significantly enhanced the migratory and invasive abilities of HCT116 and LOVO cells. To summarize, our findings demonstrate that PMP2 plays a role in inhibiting tumor metastasis during EOCRC tumorigenesis.

## 4 Discussion

The main factor for poor prognosis in CRC patients is metastasis. For example, studies have shown that the 5-year survival rate for metastatic CRC patients is only about 6% (Siegel et al., 2020; Kasi et al., 2019). Patients with EOCRC often present features, such as late-stage diagnosis, poor treatment outcomes, and unfavorable prognosis. Moreover, EOCRC exhibits stronger invasiveness, including faster growth rates and increased metastasis, leading to cancer progression to an advanced stage in a relatively short period (Hofseth et al., 2020; Mauri et al., 2018; Marx et al., 2023). Despite receiving more aggressive treatments, including surgery, chemotherapy, and radiotherapy, tumor metastasis remains the main factor influencing patient survival (Ren et al., 2024). Therefore, searching for molecular markers associated with metastasis may provide novel approaches for treating patients with EOCRC.

In this study, we used WGCNA to cluster genes with similar expression patterns into the same modules. Subsequently, gene modules related to metastasis in EOCRC and LOCRC were screened and further analysis identified genes specifically associated with EOCRC metastasis. The subsequent DO enrichment analysis revealed that the identified genes are mainly associated with Lewy body dementia and mood disorders. The GO enrichment analysis indicated that these genes are predominantly linked to synaptic organization, neuronal cell bodies, and ion channel activity. Further KEGG enrichment analysis showed that these genes are related to neuroactive ligand-receptor interaction and calcium signaling pathways. We selected four feature genes after intersecting the results from two machine learning methods; the ROC curves demonstrated the accuracy of these feature genes. Additionally, immune cell correlation analysis and immune checkpoint analysis performed to provide deeper insights into the underlying mechanisms of these feature genes in immunotherapy suggested their potential value immunotherapy targets. Our study is the first to show that PMP2 expression is downregulated in clinical EOCRC tissue samples. Additionally, PMP2 knockdown significantly enhanced the migration and invasion capabilities of HCT116 and LOVO cells, indicating that PMP2 may function as a suppressor of tumor metastasis during EOCRC progression.

This study identified four feature genes associated with EOCRC metastasis. Among these, LINC02268 was strongly associated with the survival outcomes of patients with renal clear cell carcinoma. Further, LINC02268 could activate the interleukin-6 (IL6)/janus kinase (JAK)/signal transducer and activator of transcription 3 (STAT3) signaling pathway, crucial for driving tumor advancement and metastasis in the tumor microenvironment (Wei et al., 2021; Johnson et al., 2018). The second gene AC092652.1 is a ferroptosis-related gene and may function in inflammatory bowel disease development (Tang et al., 2023). GRIK1 is the principal receptor for activating neurotransmitters in the mammalian brain, and its product is a ligand-gated ion channel, belonging to the kainate family of glutamate receptors (Chatterjee et al., 2022; Lieberman et al., 2020). According to previous studies, GRIK1 expression was decreased in CRC tissues compared to normal colorectal tissues. Moreover, GRIK1 expression was correlated with lymph node and metastatic stages, consistent with our findings in EOCRC. Additionally, CRC patients with low GRIK1 expression had shorter overall survival compared to those with high GRIK1 expression, further indicating that GRIK1 may function as a tumor metastasis suppressor in CRC (Ren et al., 2020). Many studies have indicated that GRIK1 is implicated in the occurrence of liver cancer and breast cancer through its involvement in glutamate signaling (An et al., 2018; Thursz et al., 2012; Cacace et al., 2016).

PMP2 is a small molecule lipid-binding protein of the fatty acid-binding protein (FABP) family and is closely related to lipid metabolism. Studies have shown that abnormal lipid metabolism is an important mechanism in cancer progression (Furuhashi and Hotamisligil, 2008). The expression of other members of the FABP family was correlated with tumor invasiveness and prognosis in breast cancer and prostate cancer (George Warren et al., 2023; Carbonetti et al., 2019). The PMP2 gene product is a small protein consisting of 132 amino acids and is located in the myelin of the peripheral nervous system (Baga et al., 2023). Further, PMP2 protein was shown to be involved in lipid homeostasis in the peripheral nervous system and plays an important role in intracellular lipid transport (Stettner et al., 2017; Zenker et al., 2014; Yim et al., 2022). Structural and functional disorders caused by defects in this gene are the basis of dominant demyelinating Charcot-Marie-Tooth neuropathy (Motley et al., 2016). Additionally, a study highlighted that PMP2 was involved in controlling myelin thickening and ATP synthesis during myelin regeneration (Hong et al., 2024). Another study showed that PMP2 expression in melanoma cells is regulated by the transcription factor SOX10 and is linked to melanoma cell invasivenes (Graf et al., 2019). Although the functions of PMP2 in the nervous system have been reported, no studies have yet reported a connection between PMP2 and CRC.

In this study, we could identify four feature genes associated with EOCRC metastasis by integrating WGCNA, LASSO, and SVM-RFE approaches. We also validated that the downregulation of PMP2 may be associated with poor prognosis in EOCRC patients. Our study confirmed the low expression of PMP2 in EOCRC at the molecular and protein levels. The study also demonstrated that PMP2 inhibits the metastasis of EOCRC through migration and invasion assays. This study offers interesting perspectives for future exploring EOCRC metastasis mechanisms and developing novel diagnostic biomarkers.

## Data Availability

The datasets presented in this study can be found in online repositories. The names of the repository/repositories and accession number(s) can be found in the article/supplementary material. Raw data and code for this study are available at: https://www.jianguoyun.com/p/DbSz0pAQgsGwDRiN_vMFIAA.
